# Habitat heterogeneities versus spatial type frequency variances as driving forces of dispersal evolution

**DOI:** 10.1002/ece3.1289

**Published:** 2014-11-27

**Authors:** Sebastian Novak

**Affiliations:** Institute of Science and Technology (IST) AustriaAm Campus 1, Klosterneuburg, 3400, Austria

**Keywords:** Balanced dispersal, ideal free distribution, migration, relatedness, spatial variability

## Abstract

Understanding the evolution of dispersal is essential for understanding and predicting the dynamics of natural populations. Two main factors are known to influence dispersal evolution: spatio-temporal variation in the environment and relatedness between individuals. However, the relation between these factors is still poorly understood, and they are usually treated separately. In this article, I present a theoretical framework that contains and connects effects of both environmental variation and relatedness, and reproduces and extends their known features. Spatial habitat variation selects for balanced dispersal strategies, whereby the population is kept at an ideal free distribution. Within this class of dispersal strategies, I explain how increased dispersal is promoted by perturbations to the dispersal type frequencies. An explicit formula shows the magnitude of the selective advantage of increased dispersal in terms of the spatial variability in the frequencies of the different dispersal strategies present. These variances are capable of capturing various sources of stochasticity and hence establish a common scale for their effects on the evolution of dispersal. The results furthermore indicate an alternative approach to identifying effects of relatedness on dispersal evolution.

## Introduction

The dispersal of individuals is a ubiquitous trait of any species. It embeds natural populations into their environment by setting a scale for geographic distance, and it dictates to what extent habitat heterogeneities are experienced as such or are averaged out. Furthermore, it determines the degree of admixture of a spatially structured population by providing an estimate of how many individuals interact locally. Understanding the evolution of dispersal is therefore crucial for understanding the dynamics of spatially structured populations, speciation, and the evolution of many other life-history traits. Furthermore, it helps us predict the impact of environmental change or invasions of alien species.

The propensity to disperse is variable and heritable, and hence subject to natural selection. The evolution of dispersal has attracted much interest in the past few decades, see the reviews by Bowler and Benton (2005); Dieckmann et al. (1999); Johnson and Gaines (1990); Ronce (2007). Positive dispersal must entail significant benefits, as substantial costs are associated with dispersal (Bonte et al. 2012). These costs come from the time and energy needed for dispersal, as well as from increased mortality during the dispersal phase (Johnson and Gaines 1990; Ronce 2007). In addition, local adaptation causes indirect costs for dispersers, as they are less likely to carry alleles locally favored at their destination and thus have a disadvantage in new environments (Billiard and Lenormand 2005).

Two main driving forces of dispersal evolution have been identified (Bowler and Benton 2005; Ronce 2007). First, dispersal can be seen as a mechanism to avoid competition between relatives. By reducing the relatedness, dispersal alleviates kin competition, as first proposed by Hamilton and May (1977) and studied in more detail in subsequent articles, for example, Gandon and Michalakis (1999); Rousset and Gandon (2002); Taylor (1988). Also, inbreeding depression is ameliorated by increased dispersal (Gandon 1999; Roze and Rousset 2005; Szulkin and Sheldon 2008). In practice, however, the relative impacts of inbreeding and kin competition on the evolution of dispersal are difficult to separate as both are based on the relatedness between individuals (Perrin and Goudet 2001).

Second, spatio-temporal variation of the environment interacts strongly with dispersal. If local extinction events occur, dispersal is necessary to recolonize empty habitat, and thus is maintained even if it is costly (Van Valen 1971). This is an extreme form of temporal habitat variability, which has been shown to promote dispersal (Mathias et al. 2001; Cadet et al. 2003; Bach et al. 2007; Jansen and Vitalis 2007; Blanquart and Gandon 2011; Parvinen et al. 2012). By spatial habitat heterogeneity, I refer to spatial differences in habitat quality, expressed by variable resource availability or carrying capacity, for example. In particular, I do not consider spatial heterogeneity in selection (Balkau and Feldman 1973). However, the effects of these two types of habitat heterogeneity on the evolution of dispersal are very similar: Conversely to temporal habitat variability, spatial habitat heterogeneities select against dispersal (Holt 1985; Dockery et al. 1998). Hastings (1983) argued that zero dispersal is the only evolutionarily stable dispersal strategy if the habitat is heterogeneous in space but temporally stable (see e.g., Waddell et al. (2010) for a weighting between these two kinds of variability). This is because high-quality habitat contains relatively many individuals and thus, dispersal leads to a net flux of individuals into low-quality habitat. However, Hastings pointed out that nonzero dispersal rates can be maintained under conditional (for example, density-dependent, dispersal). This idea is confirmed by McPeek and Holt (1992), demonstrating that spatial heterogeneity can select for dispersal if dispersal depends on carrying capacity. Note that at the margins of a species' range, additional factors govern the evolution of dispersal (Dytham 2009). However, in this article, I do not consider those but focus on a population that has become established within its habitat.

In the context of dispersal evolution, the ideal free distribution (Kacelnik et al. 1992) has gained significant importance. The ideal free distribution is a spatial distribution of a population with the property that individuals cannot increase their reproductive output by changing their location. As a result, all individuals have the same reproductive output, and the population is distributed as if there was no dispersal. In particular, this implies that a homogeneous population, whose growth is limited by the abundance of a fixed resource, is at its carrying capacity. Under reasonably general assumptions, dispersal strategies that lead to an ideal free distribution are evolutionarily stable (Cressman and Křivan 2006; Cantrell et al. 2007, 2010), that is, they are the expected ultimate outcomes of evolutionary trajectories. Zero dispersal as found by Hastings (1983), and the positive dispersal strategy described by McPeek and Holt (1992) are examples in support of this theory.

The dispersive ability of a population is usually characterized by its dispersal rate (migration rate) that denotes the fraction of individuals leaving their habitat patch per time unit. Classical discrete models, such as Wright's island model and the stepping stone model (Kimura and Weiss 1964), use this description of dispersal. To describe more detailed modes of dispersal, the notion of dispersal distance determines how far individuals displace from their original patch (Gandon and Rousset 1999; Murrell et al. 2002; Rousset and Gandon 2002). More generally and more commonly used in continuous models of dispersal, dispersive behavior is described by dispersal kernels. They denote probability distributions for the displacement of individuals within a time unit. A few authors have studied the evolution of whole dispersal kernels either of a fixed shape (Gros et al. 2006), or changing their shape (Hovestadt et al. 2001), mainly using numerical simulations. In the following, I present a deterministic diffusion model of type-dependent dispersal in which the mean and variance of the dispersal kernel alone determine the dispersive behavior of the population. I will denote the mean of the dispersal kernel by the mean displacement, as it describes the mean distance and direction of individual movement. The variance of the dispersal kernel I call diffusiveness. It can be interpreted as the extent to which individuals spread in space or as a measure of variability in dispersal distance among individuals. In this article, the evolution of these two determinants, mean displacement, and variance of dispersal, is studied.

## The Model

Consider a population consisting of *n* dispersal types that occupy a habitat Ω in 1-dimensional space. By *N*_*i*_(*x*,*t*) denote the densities of adults of type *i* at location *x* and time *t*, and by *p*_*i*_(*x*,*t*) their relative frequencies. *N*_*T*_(*x*,*t*) = ∑*N*_*i*_(*x*,*t*) is the total population density. Local birth and death rates of individuals are assumed to be identical for all types, and I collapse them into a single per-capita growth rate *r*(*x*,*N*_*T*_) that depends on the spatial variable *x* and the total population density *N*_*T*_. Hence, there is no direct selection on any trait. For any given position *x*, a zero of the growth rate function *r*(*x*,*N*_*T*_) = *r*_*x*_(*N*_*T*_) determines a carrying capacity *κ*_*x*_, that is, *r*_*x*_(*κ*_*x*_) = 0. Let this zero be unique to exclude, for example, strong Allee effects and let *r*_*x*_(*N*_*T*_) > 0 if *N*_*T*_ < *κ*_*x*_ and *r*_*x*_(*N*_*T*_) < 0 if *N*_*T*_ > *κ*_*x*_. Given that *r*(*x*,*N*_*T*_) is smooth, we can define a smooth carrying capacity profile *κ*(*x*) = *κ*_*x*_ for *x* ∈ Ω. In the following, I require *κ* to be strictly positive in the interior of the habitat Ω.

The dispersive behavior of each type in the population is described by a dispersal kernel *μ*_*i*_(*x*, *t*; *y*, *t* + Δ*t*), which gives the probability that an individual of type *i* located at position *x* at time *t* disperses to *y* within a short time interval Δ*t*. Let the dispersal kernels fulfill the following three assumptions, which are standard in diffusion theory. First, individuals must not move at infinite speed, that is, no finite distances can be covered in infinitesimally small time. Hence, for *ɛ* > 0 we postulate


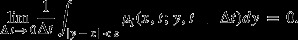
(1a)

Moreover, let the *μ*_*i*_ have (truncated) means and variances, *M*_*i*_(*x*,*t*) and *V*_*i*_(*x*,*t*), that is,


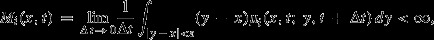
(1b)


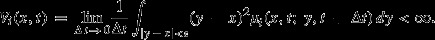
(1c)

The expected directional movement (mean displacement) and the diffusive effect of dispersal (diffusiveness) of type *i* are captured by *M*_*i*_(*x*,*t*) and *V*_*i*_(*x*,*t*), as defined in equations (1b) and (1c. If mean displacement *M*_*i*_ and diffusiveness *V*_*i*_ are constant, I speak of unconditional dispersal. Conversely, with conditional dispersal, individuals base their dispersal decisions on environmental cues such that *M*_*i*_ and *V*_*i*_ may vary in space and time. This dependence can be explicit or emerge implicitly from conditioning on, for example, the current population density or resource abundance. To indicate this – possibly indirect – spatio-temporal dependence of mean displacement and diffusiveness, I will write *M*_*i*_(·) and *V*_*i*_(·) in the case of conditional dispersal (rather than *M*_*i*_(*x*,*t*) and *V*_*i*_(*x*,*t*)).

Under the assumption that we can approximate the life cycle of reproduction followed by dispersal by a diffusion equation – namely that the population can be characterized in terms of densities, the local influences *r*(*x*,*N*_*T*_) are weak, and the *μ*_*i*_ satisfy (1), details in Appendix S1 – the dynamics of population density *N*_*T*_ and dispersal type frequencies *p*_*i*_ are given by


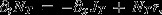
(2a)


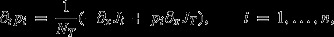
(2b)where


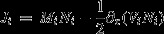
(3)

is the flux of individuals of type *i*, and *J*_*T*_ = ∑_*i*_*J*_*i*_ is the total flux of individuals. For the ease of notation, I dropped the arguments *x* and *t* throughout. Similar models have been employed by, for example, Dockery et al. (1998); Pigolotti and Benzi (2014).

The equations 2 are reaction-diffusion equations. The population disperses according to the gradient of its flux, −∂_*x*_*J*_*T*_, and is locally regulated by the per-capita growth rate *r*. I do not impose any particular regulation mechanism on population density; population regulation arises from the specification of density dependence of the growth rate *r* = *r*(*x*,*N*_*T*_). Similarly, spatial heterogeneity comes from the dependence of the growth rate on the spatial variable *x*. Interestingly, the reaction terms in the equations for the type frequencies *p*_*i*_ are determined by the total flux of individuals, ∂_*x*_*J*_*T*_. Hence, ∂_*x*_*J*_*T*_ represents a force selecting on dispersal that is detailed below. If dispersal were type-independent and unconditional (i.e., *M*_*i*_(·) ≡ *M* and *V*_*i*_(·) ≡ *V* for all *i*, and *M* and *V* constant), and *r* = *r*(*N*_*T*_) spatially homogeneous, equation (2b) simplifies to the standard diffusion equation, ∂_*t*_*p*_*i*_ = (*V*/2)∂_*xx*_*p*_*i*_. Note that from the dispersal kernels *μ*_*i*_, only *M*_*i*_ and *V*_*i*_ enter equation 2. Hence, we do not restrict to any particular shape of dispersal kernel; a dispersal strategy is characterized solely by *M*_*i*_(·) and *V*_*i*_(·).

For the equations (2), we need to specify boundary conditions. Throughout this article, I require that the habitat Ω is closed, for example, a bounded interval or a circular habitat. In the first case, no individuals must enter or leave the habitat, such that all fluxes vanish at the interval's endpoints. In the latter case, we can imagine an interval glued together at its endpoints, such that the values of all expressions, and their derivatives coincide there.

In Appendix S2, I argue that the two equations (2a) and (2b) can be separated by separating their time scales, given that the dispersal patterns of all types are sufficiently similar. Then, population density equilibrates in a rapid initial phase and can be assumed to be constant, hence ∂_*x*_*J*_*T*_ = *N*_*T*_, as type frequencies evolve on a slower time scale. In the following, I consider a resident population with a dispersal strategy characterized by mean displacement *M*_0_(·) and diffusiveness *V*_0_(·). This population is invaded by a dispersal modifier with frequency *p*_*I*_(*x*,*t*) that changes the dispersal strategy to *M*_*I*_(·) = *M*_0_(·) + *m*(·) and *V*_*I*_(·) = *V*_0_(·) + *v*(·), where *m*(·) and *v*(·) are sufficiently small. The invasion corresponds to a perturbation of the dispersal type frequencies around *p*_*I*_(*x*,*t*) = 0; the exact pattern of the perturbation (for example, local or global) is irrelevant for the long-term outcome in our continuous model. As all types at location *x* have the same growth rate *r*(*x*,*N*_*T*_), changes in modifier frequencies will be due to dispersal effects rather than different growth rates. In my study, dispersal hence does not incur any explicit cost, which could be added to the model in a straightforward way by introducing distinct growth rates *r*_*i*_(*x*, *t*) for different types, see Appendix S1, in particular equation (A7a).

## Results

I use the terminology introduced in the previous section. In addition, I denote by *N*_*I*_ the number of dispersal modifiers (invaders), and by *J*_*I*_ their flux. For the sake of improved readability, I will often omit the spatial and temporal dependence of these and similar quantities in the following. Generally, however, they will not be constant unless stated explicitly.

### Temporal change of modifier abundance

The total number of modifiers in the habitat is obtained by integrating *N*_*I*_ = *p*_*I*_N_*T*_ over the habitat Ω. Using (2), this yields


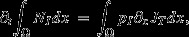
(4)

as *N*_*T*_r = ∂_*x*_*J*_*T*_ at equilibrium of *N*_*T*_. Note that integration of the flux term in (2) gives −*J*_*I*_|_Ω_, which vanishes as the habitat is closed. Equation (4) shows that the modifier will not increase in total numbers if either the total flux of individuals, *J*_*T*_, or, after partial integration, if its frequency *p*_*I*_ is constant throughout the habitat. Thus, invasion stops if the modifier's frequency spreads out evenly, but note that spatial heterogeneities in dispersal patterns or population density profiles can deform initially constant frequency profiles. Furthermore, a modifier increases if it invades regions where ∂_*x*_*J*_*T*_ is positive. As *N*_*T*_ is at equilibrium, these areas coincide with those where the growth rate *r* is positive. Thus, this finding is very natural and, in particular, does not depend on the dispersal pattern of the invading type. In general, the invader increases in numbers if the change of flux weighted by its frequency is positive. Thus, heuristically, the dispersal pattern must have the effect of keeping the invader's frequency above average in areas of positive growth rates to ensure its continuing spread.

### Ideal free distributions and stability of balanced dispersal

In the modeling section, I defined the carrying capacity profile *κ*(*x*). I call a dispersal strategy balanced (Doncaster et al. 1997) if *N*_*T*_ = *κ* is a stable solution for the dynamics of a population entirely adopting this strategy. Recalling the definition of the ideal free distribution (Kacelnik et al. 1992), a population using a balanced dispersal strategy is hence maintained at an ideal free distribution under perturbations of *N*_*T*_. From equation (2), together with (3), we see that a dispersal strategy given by *V*(·) and *M*(·) is balanced if the change in total flux, ∂_*x*_*J*_*T*_, vanishes if the population (entirely adopting it) is at carrying capacity *κ*; that is if


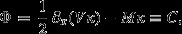
(5)

where 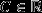 is, in particular, constant with respect to space – see also Cantrell et al. (2010). Note that an inhomogeneous composition of two or more balanced dispersal strategies at carrying capacity generally does not imply vanishing ∂_*x*_*J*_*T*_.

In Appendix S3, I prove mathematically that the class of balanced dispersal strategies is protected against invasion by (sufficiently similar) nonbalanced dispersal strategies. In this sense, balanced dispersal strategies that produce an ideal free distribution are evolutionarily stable outcomes of dispersal evolution. Evolutionary stability of balanced dispersal strategies has been shown for similar models of dispersal evolution, e.g., Cantrell et al. (2007); Cressman and Křivan (2006).

### Dynamics at ideal free distribution

Between two balanced dispersal strategies, the previous stability analysis does not provide a definite statement. In the following, I investigate dispersal evolution within the class of balanced dispersal strategies, that is, the evolution of dispersal at ideal free distribution. Assume that both the original and the modified dispersal strategies are balanced, that is, they satisfy (5). In particular, this implies that 1/2∂_*x*_*v*(*x*)*κ*(*x*) − *m*(*x*)*κ*(*x*) is constant. Replacing for the total flux *J*_*T*_, we obtain from equation (4)


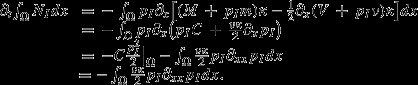
(6)

This expression is independent of the modification to mean displacement *m*. Therefore, the mean displacement does not contribute to the success or failure of the modifier as long as it adjusts a potential mismatch in diffusiveness to retain a balanced dispersal strategy.

It is remarkable that changes in diffusiveness (nonzero *v*) lead to changes in the number of modifiers as long as their frequency profile, *p*_*I*_, is not spatially constant. In full generality, the sign of this change depends on the shape of *p*_*I*_. However, if *vκ* is constant, equation (7) can be partially integrated to yield


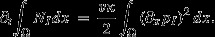
(7)

The second term from the partial integration vanishes due to the boundary conditions. This equation is analogous to equation (5) of Pigolotti and Benzi (2014), who analyzed stochastic noise in a finite population. The occurrence of equation (7) here, however, demonstrates its relevance more broadly. It shows that a growth rate of the modifier abundance proportional to *vκ* is induced if the modifier changes its diffusiveness such that dispersal stays balanced. This change is fueled by heterogeneities in the modifier's frequency, ∂_*x*_*p*_*I*_ ≠ 0. Consequently, it is only transient if the dispersal type frequency profile diffuses out over time. Thus, under a purely deterministic model without explicit costs of dispersal, or selection on a genetic background, balanced dispersal strategies are neutral with respect to each other.

However, the flattening-out of the frequency profile can be counteracted by factors not yet considered in the model, tipping the balance between the competing types. If these factors generate or maintain spatial differences in the frequency profile, they thereby make the transient effect of a variant dispersal strategy permanent. For example, selection on linked traits takes a complex role in dispersal evolution. While local adaptation is known to select against dispersal, equation (7) indicates that selective processes on a genetic background that perturb the frequency profile of dispersal modifiers, thereby can favor increased dispersal. First, selection against heterozygotes can maintain frequency heterogeneities in the form of clines (Barton 1979) in which type-dependent dispersal can operate. Note that these clines do not require spatial heterogeneity in selection but emerge, for example, after secondary contact between differentiated species. Second, transient selection patterns on a selective background linked to the dispersal modifier can directly perturb modifier frequencies away from uniformity. Third, if beneficial mutations appear on the selective background, they sweep to fixation. As recombination gradually breaks down linkage between the beneficial mutation and the dispersal modifier, the sweep has an impact on the latter's frequency profile (Barton 2000). Even though, on average, the direct effect of such sweeps – often termed "draft" (Lenormand et al. 2009) – cancels out, it hence leads to a systematic increase of modifiers that enhance dispersal.

Finally, genetic drift in finite populations perturbs type frequencies away from spatial uniformity. It has been observed that relatedness may emerge from genetic drift in a structured population (Lenormand et al. 2009). Accordingly, the variability in type frequencies due to genetic drift constitutes a measure of relatedness between individuals in the population (Barton and Clark 1990). Hence, equation (7) relates to the body of literature that dates back to Hamilton and May (1977) and predicts the promotion of positive dispersal to escape from kin competition.

## Discussion

As dispersal evolves, different dispersal strategies in a population compete against each other in a selective process. The two main factors of influence are known to be the relatedness between individuals and spatio-temporal variability of the environment. Here, by spatial heterogeneity, I referred to local differences in resource availability or carrying capacity. Other types of spatial habitat variability require additional information put into the model. For example, selection for a spatially shifting optimum requires to link dispersal to a second trait under direct selection. However, the consequences for dispersal are analogous to a variable carrying capacity: Gene flow causes individuals to be locally maladapted and hence induces a dispersal load (Kirkpatrick and Barton 1997) that enhances the pressure for lower dispersal. Historically, investigations have focused on either effects of relatedness or spatio-temporal variability of different kinds rather separately – but see, for example, Gandon and Michalakis (1999); Leturque and Rousset (2002); Morris et al. (2001); Blanquart and Gandon (2014). In this study of the evolution of dispersal, I demonstrated how the effects of environmental heterogeneity and type frequency variances, for example due to genetic drift and relatedness, can be linked within the same model.

Throughout this article, I assumed that population density is temporally constant. This can be justified if the differences in dispersal behavior between types are small. Then, population density will quickly equilibrate, and we recover a fast–slow dichotomy in which the ecological dynamics of population density can be decoupled from the dynamics of dispersal type frequencies. The assumption of small differences in dispersal strategies is reasonable if we accept that dispersal evolution proceeds in small steps. It allows us to treat population density as given while type frequencies evolve. Simulations confirm that the approximation is robust; as long as the deviations between dispersal patterns are small, simulations of the full system (2) and of ((2b) with population density *N*_*T*_ fixed produce virtually identical outcomes. In particular, however, the assumption of temporally constant population density precludes most aspects of environmental stochasticity, which is not considered in this article.

Intuitively, dispersal strategies that let the population more efficiently exploit the resources that are present in the habitat should be successful. That is, strategies that minimize the spatial discrepancies in growth rates and hence the experienced differences in habitat quality can be expected to be selectively favored. This intuition is confirmed by equation (4), which gives an analytical expression for the change of the total abundance of dispersal strategies present in the habitat. The number of individuals of a specific dispersal strategy increases if the mean derivative of the total flux, ∂_*x*_*J*_*T*_, weighted by the type's frequency, is positive. As ∂_*x*_*J*_*T*_ is proportional to the local growth rate, this result simply states that a successful type must be overrepresented in regions of positive growth rate. It follows that an evolutionarily stable dispersal strategy homogenizes the total flux *J*_*T*_, and hence equalizes local growth rates. That is, it causes the population to attain an ideal free distribution (Kacelnik et al. 1992).

In principle, this can be achieved in two ways. Zero dispersal trivially homogenizes the total flux *J*_*T*_. One of the first contributions to this aspect of dispersal evolution was by Hastings (1983), who showed that a heterogeneous environment leads to zero dispersal if dispersal is unconditional. This is because positive unconditional dispersal leads to a net flux of individuals from regions of positive growth rates (high carrying capacity) into regions of negative growth rates (low carrying capacity) and is thus to the disadvantage of the population. Accordingly, dispersal types with reduced diffusiveness exploit their environment more efficiently and therefore out-compete more mobile types. This statement is a special case of the present analysis, restricting to unconditional dispersal strategies. It has been proved earlier by Dockery et al. (1998) for a specific choice of local growth function *r*(*x*,*N*_*T*_). An illustrative description of the mechanism in a discrete setting is given by Holt (1985).

More generally, balanced dispersal strategies take the population to an ideal free distribution by matching dispersal behavior to the spatial carrying capacity profile. This class of strategies has been shown to be evolutionarily stable in previous studies, for example by Cantrell et al. (2010); McPeek and Holt (1992). If the population is at an ideal free distribution, any nonbalanced dispersal strategy changes the flux *J*_*T*_ to its own disadvantage. Accordingly, I characterized the class of balanced dispersal strategies for the present model, equation (5), and showed that it cannot be invaded by strategies from outside this class. Hence, it is evolutionarily stable and an expected long-term outcome of dispersal evolution. In practice, however, there is little empirical evidence for dispersal strategies of this type, reviewed by Diffendorfer (1998). Rather, experiments with bacteria and protozoa (Donahue et al. 2003) seem to support a source–sink dispersal type (Pulliam 1988). However, given the complexity of interaction of dispersal with other traits and the time it would take to reach an evolutionarily stable state even under controlled conditions, it is questionable if balanced dispersal is feasible to evolve in the laboratory.

Not all balanced dispersal strategies do equally well so that we can establish a selective hierarchy between them whenever dispersal type frequencies are variable in space. Analytically, this is formulated in equation (6) and, for an important special case, in equation (7). The latter shows that the total number of individuals with increased diffusiveness never declines. In fact, this number increases whenever dispersal type frequencies vary in space. In our deterministic setting, the effect levels out as frequencies diffuse in space and stalls once the frequency profiles are completely flat. In practice, however, various forces (e.g., selection on a genetic background and different sources of stochasticity) continuously perturb the frequency profiles and hence induce a variance that sustains the increase in numbers of individuals with increased diffusiveness. Thus, roughly speaking, elevated dispersal is selected for among balanced dispersal strategies.

The two forces exerted by the variability in the habitat and the variability in dispersal type frequencies can be seen as opposing each other. Spatial heterogeneity in the habitat exert a selection pressure for reduced dispersal, at least if the possibility of conditional and hence balanced dispersal is limited, as is likely the case in many natural populations. Once sufficiently close to an ideal free distribution, the variability in the dispersal type frequency profile of the population counters this force. The magnitude of the pressure for increased dispersal will depend on the balance between the size of the perturbations of frequencies away from uniformity and the homogenizing effect of dispersal.

A particular issue of dispersal evolution is whether dispersal evolves in a population that initially does not disperse at all, that is, *M*_0_ = *V*_0_ = 0. My results answer this question for the scenario studied here: Given that the population is capable of adjusting its dispersal to the geographic heterogeneities, any nonzero balanced dispersal strategy is selectively favored over the zero dispersal strategy, as long as dispersal type frequencies are variable in space.

Spatial heterogeneities in the type frequencies can emerge due to many reasons. If the type frequencies fluctuate because of genetic drift, the variance in type frequencies constitutes a measure of relatedness (Barton and Clark 1990). The fact that relatedness selects for dispersal in finite populations is well-known (Gandon and Michalakis 1999; Billiard and Lenormand 2005; Roze and Rousset 2005). Equation (7) demonstrates an alternative approach to identifying effects of relatedness in dispersal evolution via type frequency variances emerging from stochastic sampling. To illustrate how the effects of kin competition and genetic drift relate to spatial heterogeneities in type frequencies, briefly consider two examples.

First, consider a simple two-patch model with different patch sizes. In a classical paper, McPeek and Holt (1992) showed that balanced dispersal strategies, which cause the number of emigrants to equal the number of immigrants in each patch, are evolutionarily stable. Extending this model to finite populations, Leturque and Rousset (2002) defined a fitness measure taking relatedness into account. In this case, a single dispersal strategy is selected for, which both is balanced and leads to panmixia, that is, the population behaves as if mating happened randomly in a single mating pool. Assume that the population consists of two types of identical clones, one of which is present at frequencies *p*_*A*_ and *p*_*B*_ in patches *A* and *B*. Then, the quantity *χ* = (*p*_*A*_ − *p*_*B*_)^2^ is a measure of type frequency variability between the two patches, analogous to (∂_*x*_*p*_*I*_)^2^ in equation (7). One can easily show that *χ* is minimized for panmixia with *χ* = 0, hence dispersal increases as long as this quantity is positive and equilibrates when *χ* = 0.

The variability of type frequencies between the patches thus plays an interesting role and could be used as a measure for the benefit of dispersal in alleviating kin competition in this example.

Second, one could incorporate genetic drift directly into the model (2). This has been done by Pigolotti and Benzi (2014), who obtained equation (7) from their resulting stochastic partial differential equation. However, to evaluate this quantity, they had to introduce a (spatial) cutoff *ε*, which is hard to interpret biologically. Considering a stepping stone model (Kimura and Weiss 1964) as a discrete version of the continuous model, (2) shows that the expected change in the total abundance, 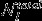, of a dispersal modifier that increases the migration rate between patches from 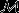 to 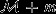 is given by


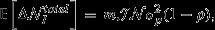
(11)

(cf. Appendix S4 for details) where 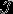 is the number of patches the habitat consists of, and 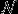 is the number of individuals present in each patch. Furthermore, 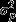 denotes the spatial variance of type frequencies, and *ρ* is the correlation between type frequencies in adjacent patches. The expression 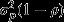 is the discrete-space equivalent to (∂_*x*_*p*_*I*_)^2^ in equation (7). The fact that 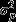 is a measure of relatedness was already noted by Pigolotti and Benzi (2014). Driving the analysis further (cf. Appendix S4), one can derive a selection coefficient for dispersal modifiers as 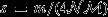. This shows that the cutoff in the article by Pigolotti and Benzi (2014) needs to be chosen as 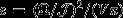, where 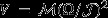, to establish the correspondence between a discrete stepping stone model and its approximation, the diffusion model (2). Hence, a possibility for measuring the selective benefit of dispersal modifiers due to relatedness is provided by the present framework.

Overall, the spatial heterogeneities of type frequencies take a central role in translating stochastic effects into selective forces promoting dispersal. Previous studies developed rather specialized models to analyze the impact of different stochastic factors on the evolution of dispersal. Direct methods are crucial for understanding the detailed process of how they influence dispersal evolution, but make it difficult to compare their relative importance. However, these stochastic factors are reflected in the same variability of type frequencies. Thus, their mode of promoting increased dispersal is channeled through the same phenomenon, as noted already by Waddell et al. (2010). Identifying their contributions to the variability of type frequencies hence puts these stochastic factors on a single scale.

In summary, my study shows that many of the main factors of dispersal evolution can be brought together in a single modeling framework. The effect of spatially varying resource availability and the consequent spatial density variations are phrased in terms of the fluxes *J*_*I*_ and *J*_*T*_. Environmental stochasticity is not considered in this article, but could be implemented directly into the equation for the total population size, equation (2a). Genetic drift and relatedness are reflected in the variability of dispersal type frequencies, (∂_*x*_*p*_*I*_)^2^, that exerts a selection pressure for increased dispersal. In many cases, selection on a genetic background can lead to heterogeneities in dispersal modifier frequencies, for example in hybrid zones, if selection transiently favors a certain part of the population, or by sweeping beneficial alleles. Indirectly, selection on a genetic background hence can also exert a positive selection pressure on dispersal modifiers that is channeled through the spatial variability of type frequencies. On top of that, dispersal evolution is limited by direct costs of dispersal in practice, which can be added to the model straightforwardly by introducing distinct growth rates *r*_*i*_(*x*, *t*) ≠ *r*_*j*_(*x*, *t*) for different types *i* and *j*. This is indicated in Appendix S1, but I did not consider direct costs of dispersal otherwise.

The results described in this article suggest that future studies should focus on the variability of type frequencies as a force promoting increased dispersal and establish its connection to demographic and environmental stochasticity more closely. I argued that selective pressures on traits linked to dispersal may maintain spatial patterns that dispersal differences can act on. The complexity of interactions between selection and type-dependent dispersal is hard to assess, but can be relevant in nature, in particular if individuals base dispersal decisions on their fitness. The correlations between fitness and dispersal are virtually unexplored, and it is unclear to what extent the ability to detect and interpret fitness conditions can be based on a genetic level. In the presence of density-dependent selection, type-dependent dispersal might tip the balance by pushing population density above thresholds and lead to interesting phenomena.

## Supporting Information

**Appendix S1.** Derivation of the model equations. **Appendix S2.** Separation of time scales. **Appendix S3.** Stability of balanced dispersal. **Appendix S4.** Genetic drift in a stepping stone model.
