# Supplementary material for: Habitat heterogeneities versus spatial type frequency variances as driving forces of dispersal evolution
Source: Ecol Evol. 2014 Nov 27;4(24):4589–97. doi: 10.1002/ece3.1289 (PMC4278812; doi:10.1002/ece3.1289)
Supplement: Supplementary file 1 [file ece30004-4589-sd1.pdf]

Appendix S1 Derivation of the model equations

Here, I derive the model equations (2) for a population consisting of n types that occupy a habitat $\Omega \subseteq \mathbb{R}$. In addition to the main text, I consider variable growth rates between types to illustrate how, e.g., explicit cost of dispersal or selection could be incorporated into the model. Let $r_i = r_i(x, N_T)$ denote the per-capita growth rate of type i.

Local reproduction changes the type densities N_i to N_i^*. We assume that this change is small, namely proportional to the (infinitesimal) time interval under consideration, Δt, and neglect all weaker effects, $o(\Delta t)$. Then, we can write

$$N_i^*(x,t) = N_i(x,t)(1 + r_i(x,t)\Delta t) + o(\Delta t). \tag{A1}$$

Second, we model dispersal. For each individual of type i that is located at position y at time t, the probability to migrate into an interval around x of length Δx within Δt time units is expressed via the dispersal kernels μ_i by $\mu_i(y,t;x,t+\Delta t)\Delta x$. Using the dispersal kernels μ_i, which naturally fulfil $\int_\Omega \mu_i(y,t;x,s)dx = 1$, we obtain the next generation by

$$N_i(x, t+\Delta t) = \int_\Omega N_i^*(y,t)\mu_i(y,t;x,t+\Delta t)dy. \tag{A2}$$

If the dispersal kernels fulfil the assumptions (1) and are sufficiently smooth, they satisfy a Kolmogorov forward equation (see, e.g., Bharucha-Reid (1960), pp.130–136)

$$\begin{aligned}\partial_s \mu_i(y,t;x,s) =& \frac{1}{2}\partial_{xx}\left(V_i(x,t)\mu_i(y,t;x,s)\right) - \\ &- \partial_x\left(M_i(x,t)\mu_i(y,t;x,s)\right).\end{aligned} \tag{A3}$$

Using equations (A1) and (A2), and dividing by Δt, we find

$$\begin{aligned}&\frac{N_i(x,t+\Delta t) - N_i(x,t)}{\Delta t} = \\ &= \int_\Omega N_i(y,t)\ \frac{\mu_i(y,t;x,t+\Delta t) - \mu_i(y,t;x,t)}{\Delta t}\ dy\ + \\ &+ \int_\Omega \left(N_i(y,t)r_i(y,t) + \frac{o(\Delta t)}{\Delta t}\right)\mu_i(y,t;x,t+\Delta t)\ dy.\end{aligned} \tag{A4}$$

For $\Delta t \to 0$, (A3) can be inserted into (A4) to obtain

$$\begin{aligned}\partial_t N_i(x,t) = & \frac{1}{2}\partial_{xx}\left(V_i(x,t)\int_\Omega N_i(y,t)\mu_i(y,t;x,t)dy\right) - \\ &- \partial_x\left(M_i(x,t)\int_\Omega N_i(y,t)\mu_i(y,t;x,t)dy\right) + \\ &+ \int_\Omega N_i(y,t)r_i(y,t)\mu_i(y,t;x,t)dy.\end{aligned} \tag{A5}$$

Since $\mu_i(y,t;x,t)$ is a point mass, the integrals resolve to

$$\partial_t N_i = -\partial_x J_i + N_i r_i, \tag{A6}$$

where $J_i = M_i N_i - \frac{1}{2}\partial_x(V_i N_i)$ is the flux of individuals of type i as in the main text.

With $N_T(x,t) = \sum_i N_i(x,t)$ and $p_i(x,t) = \frac{N_i(x,t)}{N_T(x,t)}$, a short calculation shows that N_T evolves according to

$$\begin{aligned}\partial_t N_T &= -\partial_x \left(\bar{M} N_T - \frac{1}{2}\partial_x \left(\bar{V} N_T\right)\right) + \sum_{j=1}^{n} N_j r_j \\ &= -\partial_x J_T + N_T \bar{r},\end{aligned} \tag{A7a}$$

where $\bar{r} = \sum_{j=1}^{n} p_j r_j$, $\bar{M} = \sum_{j=1}^{n} M_j p_j$ and $\bar{V} = \sum_{j=1}^{n} V_j p_j$, and $J_T = \sum_i J_i$ is the overall flux of individuals. Applying the quotient rule to $\partial_t p_i = \partial_t (N_i/N_T)$ furthermore gives

$$\begin{aligned}\partial_t p_i &= \frac{1}{N_T}\left(-\partial_x J_i + p_i \partial_x J_T + N_i r_i - p_i \sum_{j=1}^{n} N_j r_j\right) \\ &= \frac{1}{N_T}\left(-\partial_x J_i + p_i \partial_x J_T\right) + p_i (r_i - \bar{r}).\end{aligned} \tag{A7b}$$

Compare (A7b) to the model by Nagylaki & Moody (1980). With $r_i = r$ for all i, equations (A7) simplifies to the model equations (2).

Appendix S2 Separation of time scales

Assume that growth rates and dispersal patterns are identical for all types, i.e., $r_i \equiv r$, $M_i \equiv M_0$ and $V_i \equiv V_0$ for all i. We write the dynamics of this reference population $N_{0,T}$ as

$$\partial_t N_{0,T} = -\partial_x J_{0,T} + r N_{0,T}, \tag{A8}$$

where the reference flux is given by $J_{0,T} = M_0 N_{0,T} - 1/2 \partial_x V_0 N_{0,T}$. Now suppose that dispersal patterns deviate only slightly from the reference pattern, $M_i = M_0 + m_i$ and $V_i = V_0 + v_i$, where m_i and v_i (and their derivatives) are of order $O(\varepsilon)$. Furthermore assume that $\partial_x p_i$ and $\partial_{xx} p_i$ stay bounded. This assumption is natural since local agglomerates flatten out by diffusion. Then, the population density dynamics, equation (2a), are dominated by the reference dynamics (A8), i.e., they differ only up to order $O(\varepsilon)$:

$$\begin{aligned}\partial_t N_T =& -\partial_x J_{0,T} + r N_T + \frac{\bar{v}}{2}\partial_{xx} N_T - \\ & - (\bar{m} - \partial_x \bar{v})\partial_x N_T - \left(\partial_x \bar{m} + \frac{\partial_{xx}\bar{v}}{2}\right) N_T \\ =& -\partial_x J_{0,T} + r N_T + O(\varepsilon),\end{aligned} \tag{A9}$$

where $\bar{m} = \sum m_j p_j$ and $\bar{v} = \sum v_j p_j$ are the average deviations from the reference values M_0 and V_0. Since $r_i \equiv r$ for all i, the right hand side of equation (2b) can be approximated by the reference flux as

$$\partial_t p_i \approx \frac{1}{N_T}\left(-\partial_x J_{0,i} + p_i \partial_x J_{0,T}\right), \tag{A10}$$

where $J_{0,i} = M_0 p_i N_{0,T} - 1/2 \partial_x V_0 p_i N_{0,T}$. Assuming that type frequency clines spread out such that $\partial_x p_i$ and $\partial_{xx} p_i$ become negligibly small, expanding the right hand side of (A10) shows that $\partial_t p_i = O(\varepsilon)$.

Appendix S3 Stability of balanced dispersal

Consider the dynamics of two types in terms of (A6) for uniform growth rates $r_0 = r_I = r$,

$$\partial_t N_0 = -\,\partial_x J_0 + N_0 r, \tag{A11a}$$

$$\partial_t N_I = -\,\partial_x J_I + N_I r, \tag{A11b}$$

and assume that the original type N_0 follows a balanced dispersal strategy, i.e., $\partial_x V_0\kappa - M_0\kappa = const$. The dispersal strategy of the modified type N_I deviates from that of N_0 only slightly, $V_1 = V_0 + v$ and $M_1 = M_0 + m$. In the absence of the modifier, N_0 equilibrates at carrying capacity $N_0^* = \kappa$. I show that this equilibrium is asymptotically stable against invasion of non-balanced dispersal strategies.

At equilibrium, $r \equiv 0$. Setting $N_I(x,t) = \kappa e^{-\lambda t} Q(x)$, equation (A11b) transforms into an eigenvalue problem of Sturm-Liouville type (Courant & Hilbert, 1954; Weinstock, 1974):

$$0 = \frac{1}{2}\,\partial_x\left((V_0+v)\kappa\partial_x Q\right) + \partial_x(\Phi Q) + \lambda\kappa Q, \tag{A12}$$

where $\Phi = 1/2\partial_x(v\kappa) - m\kappa$. Upon multiplication with the integrating factor

$$\frac{2e^{\xi}}{(V_0+v)\kappa}, \qquad \text{where } \xi = \int \frac{\partial_x[(V_0+v)\kappa] + \Phi}{(V_0+v)\kappa}dx,$$

equation (A12) can be written in self-adjoint form

$$0 = \partial_x\left(e^{\xi}\partial_x Q\right) + \left(2e^{\xi}\frac{\partial_x\Phi}{(V_0+v)\kappa} + \lambda\frac{2e^{\xi}}{V_0+v}\right)Q. \tag{A13}$$

If Φ is constant, the eigenvalues of (A13) are known to constitute a non-negative sequence $0 \le \lambda_0 < \lambda_1 < \ldots$ (Weinstock, 1974). It is easy to see that $\lambda_0 = 0$ is an eigenvalue with constant eigenfunction $Q_0 \equiv const$. Therefore, if the modifier uses a balanced dispersal strategy ($\Phi \equiv const$), it is neutral with respect to the original type.

Now assume that $\Phi \neq const$. If m and v are small, $\partial_x\Phi$ is small and equation (A13) can be seen as a perturbation of the case $\Phi \equiv const$. Eigenvalues of (A13) depend continuously on the coefficients of the system (Courant & Hilbert, 1954). Thus, the following perturbation analysis is justified. I write $m = \varepsilon\tilde{m}$ and $v = \varepsilon\tilde{v}$. Then, $\Phi = \varepsilon\tilde{\Phi}$ and we consider the smallest eigenvalue and its eigenfunction to be a function of ε, i.e., $\lambda_0 = \lambda_0(\varepsilon)$ and $Q_0 = Q_0(\varepsilon)$. With this notation, the derivative $d\lambda_0/d\varepsilon$ determines the stability of the system.

Using the variational characterization of eigenvalues of (A13), $Q_0(\varepsilon)$ minimizes the functional

$$\int_\Omega e^{\xi}(\partial_x Q)^2 - \varepsilon\,\frac{2e^{\xi}\partial_x\tilde{\Phi}}{(V_0+v)\kappa}\,Q^2 dx, \tag{A14}$$

and its minimal value is $\lambda_0(\varepsilon)$. Since $\lambda_0(0) = 0$ and $Q_0(0) \equiv const$, the required derivative is

$$\frac{d\lambda_0}{d\varepsilon}(0) = \int_\Omega \frac{2\Phi^2}{(V_0+v)\kappa}e^{\int \Phi/[(V_0+v)\kappa]}dx - \left(e^{\int \Phi/[(V_0+v)\kappa]}\Phi\right)\Big|_\Omega. \tag{A15}$$

This expression is positive since the last term vanishes under reasonable assumptions on the boundaries of the habitat. Thus, the introduction of a non-balanced dispersal strategy causes the minimal eigenvalue to become positive and hence, the original population is protected from invasion by a non-balanced dispersal strategy.

Appendix S4 Genetic drift in a stepping stone model

We employ a stepping stone model (Kimura & Weiss, 1964) as a discrete analogue of the type frequency dynamics under type-dependent dispersal, equation (2b). The recursion for the type frequencies reads

$$p'_{(j)} = \frac{p_{(j)} + \frac{\mathcal{M}+m}{2}\left(p_{(j-1)} + p_{(j+1)} - 2p_{(j)}\right)}{1 + \frac{m}{2}\left(p_{(j-1)} + p_{(j+1)} - 2p_{(j)}\right)} \qquad j = 1, ..., \mathcal{J}, \tag{A16}$$

where the prime denotes frequencies measured in the next generation. With this notation, I assume that the habitat Ω consists of $\mathcal{J}$ equally spaced patches. Dispersal is described by migration rates $0 < \mathcal{M} < 1$ that determine the fraction of individuals leaving their patch to migrate to one of the adjacent patches (nearest-neighbour migration) with equal probability. These rates then translate into diffusiveness values of $V = \mathcal{M}(\Omega/\mathcal{J})^2$ and $M = 0$. The modifier type has frequency $p_{(j)}$ in patch j and migration rate $\mathcal{M} + m$, the original type thus has frequency $1 - p_{(j)}$ and migration rate $\mathcal{M}$.

If each of the $\mathcal{J}$ patches contains $\mathcal{N}$ individuals, the total number of individuals present in the habitat is $\Omega N_T = \mathcal{J}\mathcal{N}$. However, population size does not enter equation (A16) since it is assumed to be constant. This assumption can be justified by a specific population regulation mechanism or by taking m sufficiently small. Note that carrying capacity is considered to be spatially homogeneous and dispersal to be unconditional, hence population size is at carrying capacity at all times. Starting out from equation (A16), we can derive an expression for the change in the number of modifiers during one generation. For $m \ll \mathcal{M} \ll 1$, it reads

$$\begin{aligned}\Delta\mathcal{N}_I^{total} &= \sum_j \mathcal{N}\left(p'_{(j)} - p_{(j)}\right) = \\ &= -\frac{m\mathcal{N}}{2}\sum_j p_{(j)}\left(p_{(j-1)} + p_{(j+1)} - 2p_{(j)}\right).\end{aligned} \tag{A17}$$

This expression is equivalent to a discretization of the corresponding equation in the continuous setting, equation (7). If the habitat is homogeneous, the system is translation invariant at equilibrium. Then, taking the expectation of (A17) over a realization of the sampling process, we find that the expected change in total modifier abundance is given by equation (8).

The variance σ_p^2 and the between-patch correlation ρ of type frequencies in space have been analysed under dispersal and selection, e.g., Felsenstein (1975); Nagylaki (1978), and under dispersal and mutation, e.g., Kimura & Weiss (1964); Weiss & Kimura (1965), ultimately yielding expressions for these quantities at stochastic equilibrium. Additional mechanisms like selection or mutation need to be evoked, since under random drift alone one type will eventually fix in the population, which leads to zero variance of type frequencies at equilibrium. In the articles mentioned above, only the presence of a single dispersal type in the population has been analysed. However, since the migration modification m is small, we will expand (8) only up to leading order in m such that this deficiency can be ignored.

I follow Kimura & Weiss (1964) to derive the variance of type frequencies, σ_p^2, and the correlation between type frequencies in adjacent patches, ρ. For the purpose of this calculation, I assume an infinite habitat where patches are indexed by $j \in \mathbb{Z}$. Let $\mathfrak{M}$ denote the migration matrix, i.e., $\mathfrak{M}_{ij}$ is the migration rate from patch i to patch j. For nearest-neighbour migration set $\mathfrak{M}_{ij} = 1 - \mathcal{M}$ for $i = j$, $\mathfrak{M}_{ij} = \mathcal{M}/2$ for $j = i \pm 1$, and $\mathfrak{M}_{ij} = 0$ in all other cases. Denote

the mutation rates to and from the focal type by ν_1 and $\nu_2 = \nu - \nu_1$, such that $\nu = \nu_1 + \nu_2$ is the total mutation rate. The dynamics of type frequencies $p_{(j)}$ is then given by

$$p'_{(j)} = (1 - \nu)\,(\mathfrak{M}.\mathbf{p})_j + \nu_2 + \xi_j \tag{A18}$$

where $\mathbf{p} = (p_{(j)})_{j\in\mathbb{Z}}$ summarizes local type frequencies in a single vector. The random variable ξ_j describes genetic drift independently in each patch. It has zero mean and a variance of $p_{(j)}(1 - p_{(j)})/(2\mathcal{N})$.

From this, we can derive recursions for the expected type frequency, its variance and the covariance between patches – see also Fleming & Su (1974). Expected type frequencies settle at their homogeneous equilibrium determined solely by the mutation rates. Hence, I set $\mathbb{E}[p_{(j)}] = P$ uniformly in space. The remaining equations for the variances and covariances of gene frequencies can be solved assuming that correlations between patches decay geometrically with distance. For $\nu \ll \mathcal{M} \ll 1$ we obtain

$$\sigma_p^2 = \frac{P(1-P)}{1 + 4\mathcal{N}\sqrt{2\mathcal{M}\nu}} \quad \text{and} \quad \rho = 1 - \frac{\sqrt{2\mathcal{M}\nu}}{\mathcal{M}}, \tag{A19}$$

see Kimura & Weiss (1964). Inserting into equation (8) produces

$$\mathbb{E}\left[\Delta\mathcal{N}_I^{total}\right] = m\mathcal{I}\mathcal{N}\frac{P(1-P)\sqrt{2\mathcal{M}\nu}}{\mathcal{M}(1 + 4\mathcal{N}\sqrt{2\mathcal{M}\nu})}. \tag{A20}$$

If the number of individuals per patch, $\mathcal{N}$, is large, the result simplifies as the denominator is approximately $4\mathcal{N}\mathcal{M}\sqrt{2\mathcal{M}\nu}$. Then, we can rewrite equation (A20), as

$$\mathbb{E}\left[\Delta\mathcal{N}_I^{total}\right] = \frac{m\mathcal{I}}{4\mathcal{M}}P(1-P).$$

Denote the average frequency of the modifier in the habitat by P. Then, ividing by the total population size $\mathcal{I}\mathcal{N}$, we obtain

$$\mathbb{E}\left[\Delta P\right] \approx \frac{m}{4\mathcal{N}\mathcal{M}}P(1-P). \tag{A21}$$

This expression is analogous to a haploid selection model with selection parameter $s = m/(4\mathcal{N}\mathcal{M})$.

References

Bharucha-Reid, A.T. 1960. *Elements of the theory of Markov processes and their applications.* McGraw-Hill Series in Probability and Statistics. McGraw-Hill Book Co., Inc., New York.

Courant, R. & Hilbert, D. 1954. *Methods of Mathematical Physics, Vol. I.* Interscience, New York.

Felsenstein, J. 1975. Genetic drift in clines which are maintained by migration and natural selection. *Genetics* **81**: 191–207.

Fleming, W.H. & Su, C.H. 1974. Some one-dimensional migration models in population genetics theory. *Theoretical Population Biology* **5**: 431–449.

Kimura, M. & Weiss, G.H. 1964. The stepping stone model of population structure and the decrease of genetic correlation with distance. *Genetics* **49**: 561.

Nagylaki, T. 1978. Random genetic drift in a cline. *Proceedings of the National Academy of Sciences* **75**: 423–426.

Nagylaki, T. & Moody, M. 1980. Diffusion model for genotype-dependent migration. *Proceedings of the National Academy of Sciences* **77**: 4842–4846.

Weinstock, R. 1974. *Calculus of Variations.* Dover, New York.

Weiss, G.H. & Kimura, M. 1965. A mathematical analysis of the stepping stone model of genetic correlation. *Journal of Applied Probability* pp. 129–149.
